# Effect of Intracapsular Pressure on Decompression Effectiveness

**DOI:** 10.1016/j.identj.2022.06.023

**Published:** 2022-08-30

**Authors:** Xianbin Xiong, Changshun Li, Li Guo, Yuanyuan Wu, Yi Wang, Xiaomeng Song

**Affiliations:** a Key Laboratory of Oral Diseases of Jiangsu Province and Stomatological Institute of Nanjing Medical University, Nanjing Medical University, Nanjing, China; b Department of Oral and Maxillofacial Surgery, Affiliated Stomatological Hospital, Nanjing Medical University, Nanjing, China; c Department of Affiliated Stomatological Hospital of Xuzhou Medical University, Xuzhou, Jiangsu, China

**Keywords:** Intracapsular pressure, Decompression, Jaw cystic lesions, IL-1α

## Abstract

**Aim:**

The objective of this research was to analyse the correlation between intracapsular pressure and shrinkage rate of cystic lesion volume at different time points after decompression and to evaluate the relationship between the concentration of interleukin-1α (IL-1α) in cystic fluid and intracapsular pressure.

**Methods:**

Fifty patients with jaw cystic lesions who underwent decompression were included. We measured the intracapsular pressure and IL-1α concentration in the cyst fluid. Moreover, we calculated the rate of shrinkage (RS) of cystic cavity volume at different time points. In addition, data on age, sex, preoperative cystic cavity volume, and lesion location were collected. Linear correlation analysis and variance analysis were used for statistical analysis.

**Results:**

Fastest volume decline was observed between 0 and 3 months after surgery; the average RS_0–3_ was 45.71%. RS_3-6_ presented the second-fastest volume decline, with an average of 17.46%, and RS_6-12_ presented the slowest volume decline, with an average of 3.933%. A statistically significant difference in RS was observed amongst the 3 time points (*P* < .0001). RS_0-3_ was negatively correlated with intracapsular pressure (*r* = −0.6326, n = 50, *P* < .0001). A negative correlation between the preoperative cystic cavity volume and intracapsular pressure (*r* = −0.6384, n = 50, *P* < .001) was also observed. A significant positive correlation was observed between preoperative cystic cavity volume and RS_0-3_ (*r* = 0.611, n = 50, *P* < .0001). Moreover, a significant positive correlation was observed between the intracapsular pressure and IL-1α concentration in the cystic fluid (*r* = 0.03477, n = 50, *P* < .0001).

**Conclusions:**

Intracapsular pressure and the preoperative volume were the factors that affected the RS during the first 3 months after surgery. Therefore, the effectiveness of decompression can be evaluated by the intracapsular pressure and preoperative volume.

## Introduction

Jaw cystic lesions are diseases with clinical and imaging manifestations of cystic changes, such as jaw tumours and jaw cysts, that affect oral and maxillofacial regions. Surgery is the first-line treatment for maxillary cystic lesions. At present, the surgical methods for treating cysts mainly include curettage, decompression by fenestration, and extensive resection of cystic lesions.

For large-sized jaw cystic lesions that affect the adjacent tissue structures, such as teeth, inferior alveolar nerve, and maxillary sinus, decompression can be performed to shrink the cystic cavity, promote osteogenesis, and effectively protect the adjacent tissues.^1^^,^^2^ After decompression, a new inferior alveolar nerve tube can be formed by inferior alveolar nerve transposition; this procedure may return the electrical activity of the dental nerve to normal, thereby avoiding root canal treatment.^3^, ^4^, ^5^ Regarding curettage, secondary curettage results in a significantly lower recurrence rate than direct curettage.^6^^,^^7^

Jaw cystic lesion progression is closely related to intracapsular pressure, which is the main cause of direct or indirect jaw erosion.^8^^,^^9^ An in vitro experiment demonstrated that mechanical stress induces osteoclast formation, activates osteoclasts directly or indirectly, and stimulates bone resorption.^10^ The osteoclast content is closely related to the inflammatory factors (interleukin-1, prostaglandin [PG], etc) in the cystic fluid and the intracapsular pressure.^11^^,^^12^

The most studied risk factors for jaw cysts include pathologic type, age, and pathologic size. The effect of intracapsular pressure on postoperative osteogenesis rate has been rarely reported by both local and foreign studies. It is crucial to determine the amount and speed of bone formation at different time points after the fenestration of jaw cystic lesions. Therefore, this article analysed the relationship between intracapsular pressure and the degree of capsular volume reduction and interleukin-1α (IL-1α) concentration at different time points after fenestration.

## Materials and methods

Fifty patients with jaw cystic lesions diagnosed based on imaging and pathologic examinations at the Affiliated Stomatological Hospital, Nanjing Medical University, from April 2018 to October 2021 were enrolled in this study. There were 11 cases of odontogenic keratocyst, 22 cases of radicular cysts, 11 cases of ameloblastoma, and 6 cases of follicular cysts. This project was approved by the ethics board of the Affiliated Stomatological Hospital of Nanjing Medical University (PJ2018–043–001). Written informed consent was obtained from all patients.

The inclusion criteria were as follows: The postoperative pathology confirmed the diagnosis as jaw cystic lesions. No infection occurred in the jaw cystic lesions. No capsular wall damage was detected in the jaw cystic lesions. Monocular bone cystic lesions are present. The exclusion criteria were a solid tumour in the cystic cavity of the jaw bone. Polycystic lesions are present in the jaw bone. Maxillary cystic lesions that were not treated surgically are present.

### Measurement of pressure sensors and collection of cystic fluid

**Fig. 1:**
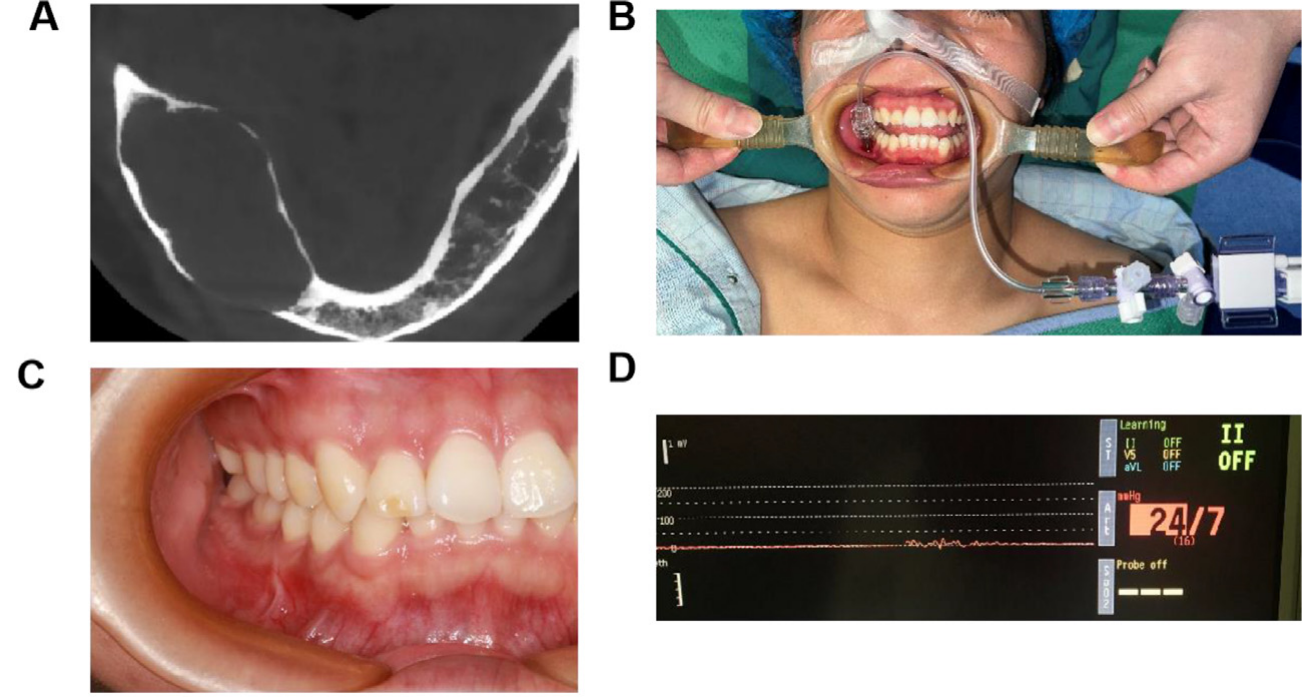
Measurement of intracapsular pressure. A, Imaging findings of the cystic lesion. B, Intraoral photograph of intracapsular pressure measurement. C, Intraoral photograph of the cystic lesion. D, The value of intracapsular pressure on the oscilloscope.

A disposable arterial pressure monitoring kit containing pressure sensors and polyethylene pipes was prepared for each patient. One end of the pressure sensor was connected to the arterial blood pressure monitoring interface of the anesthesia machine, and a 20-gauge needle was connected to the free end of the other side. Normal saline was slowly injected into the polyethylene tube, and the gas was completely discharged. The free end of the needle was aligned with the bone weakness area of jaw cystic lesions or the exposed area of the capsular wall after intraoperative ultrasonic bone knife osteotomy; the needle was in the same height plane as the pressure sensor. At the same time, the pressure sensor was calibrated to adjust the pressure value to 0 mm Hg. The needle was inserted into the capsule, and the value on the oscilloscope was observed. The intracapsular pressure fluctuated between 1 and 2 mm Hg; therefore, the median value was recorded, and 5 mL of capsular fluid was extracted (Figure 1).

### Surgical methods

All patients were operated on by doctors in charge or senior doctors of the Department of Oral and Maxillofacial Surgery at the Affiliated Stomatological Hospital, Nanjing Medical University. Preoperative routine maxillofacial examination and cone beam computed tomography (CBCT) were performed. Operation location was determined on the basis of the gravity of the low position, weak bone area, the requirement of tooth extraction, and other factors. Patients were divided into 2 surgical groups based on whether the intracapsular pressure before surgery had been measured. In patients for whom the preoperative measurement of intracapsular pressure was completed, bone was exposed by a conventional flap. A part of the bone wall was removed by bone chisel and the cystic wall was removed by electric knife or tissue shear, and a pathologic examination was conducted. After the pathologic results were clear, iodoform gauze strips were stuffed, and mucosa was sutured. In patients for whom the preoperative measurement of intracapsular pressure was not completed, the bone was exposed by opening the mucous membrane. A part of the bone was removed, and a small spherical part of the cystic wall was exposed by piezosurgery (attention was paid to protect the cystic wall integrity). After that, the intracapsular pressure was measured, and the cystic fluid was extracted. After surgery, iodoform gauze strips were indwelled for about 1 week in the cystic cavity, and the stopper was made by appointment. One week later, the iodoform gauze strip was removed, and the operation site was irrigated with normal saline at least 2 to 3 times a day. All patients were requested to return to the clinic at 3, 6, and 12 months postoperatively, and CBCT was performed.

### Measurement of jaw cystic lesion volume and calculation of the rate of shrinkage (RS)

**Fig. 2:**
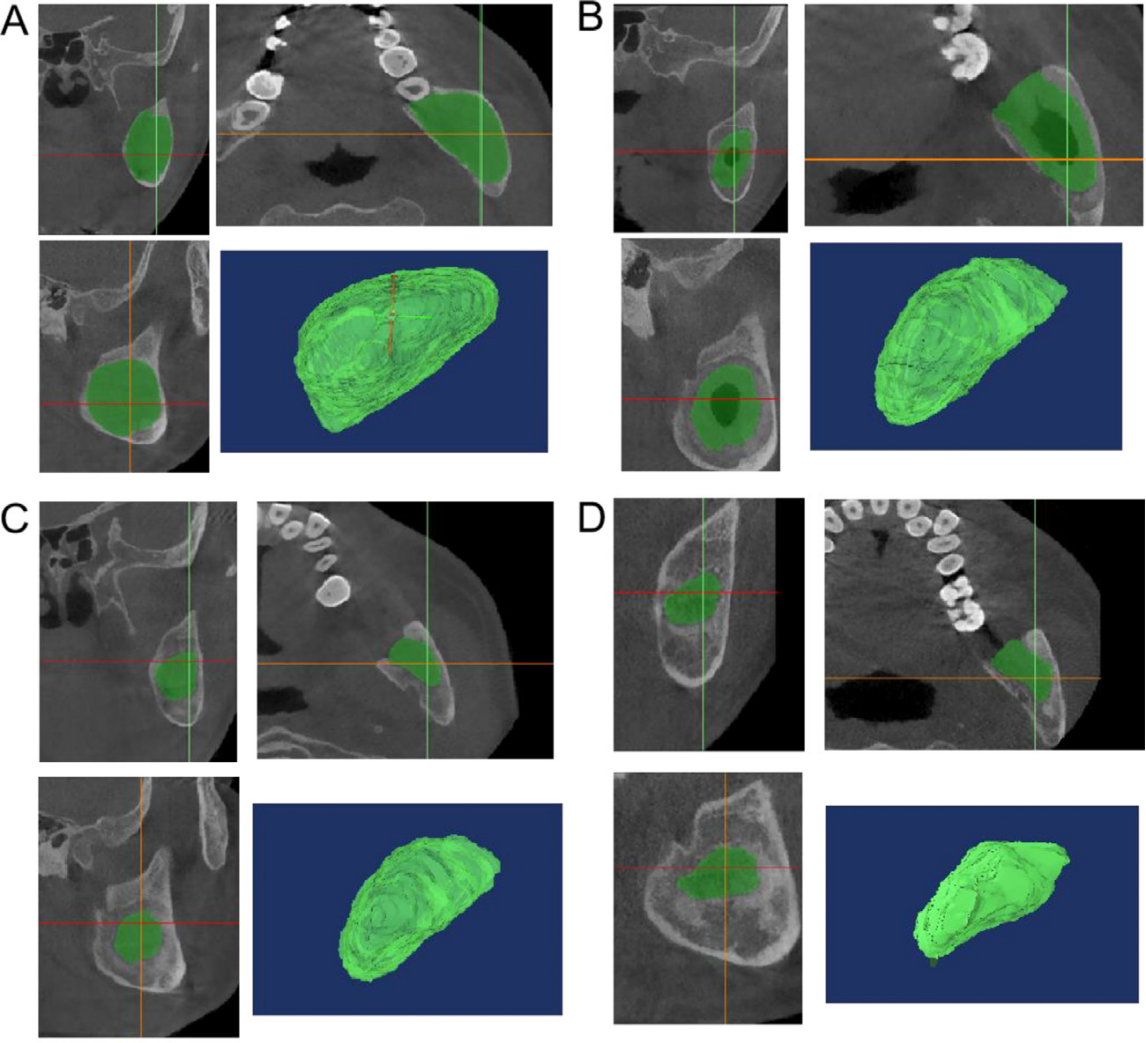
Mimics 17.0 3-dimensional reconstruction of the lumen of the same patient at different periods. A, Preoperative cavity. B, Postoperative cavity at 3 months. C, Postoperative cavity at 6 months. D, postoperative cavity at 12 months.

Mimics 17.0 was used to measure the lumen volume of each patient at different time points; subsequently, RS was calculated at each stage by using the following formula (Figure 2):

RS = difference of cystic cavity volume at different time points/preoperative cystic cavity volume/time unit × 100%

Every 3-month period was considered a time unit (RS_0–3_ =[preoperative cystic volume − cystic volume at 3 months after surgery] / preoperative cystic volume / 1  ×  100%; RS_3–6_ = [cystic volume at 3 months after surgery − cystic volume at 6 months after surgery] / preoperative volume / 1  ×  100%; RS_6–12_ = (cystic volume at 6 months after surgery − cystic volume at 12 months after surgery)/preoperative volume/2  ×  100%).

### Determination of IL-1α concentration in sac fluid

Indirect enzyme-linked immunosorbent assay (ELISA) was used to determine IL-1α concentration. The sac fluid, stored at −80 °C, was kept at room temperature for dissolving. Meanwhile, the plate containing IL-1α antigen was incubated at room temperature for 20 minutes to reach equilibrium. A blank control was set up by adding only a chromogenic agent and termination liquid. Subsequently, 50-µL capsules or standard products of different concentrations were added to the plate hole, and the plate was sealed and incubated for 120 minutes. The plate was washed 5 times and blotted with absorbent paper. Subsequently, 100 µL of antibody working solution was added to each well, and the plate was sealed and incubated for 60 min. The plate was washed 5 times and blotted with absorbent paper. Subsequently, 100 µL of enzyme-binding solution was added to each well, and the plate was incubated in the dark for 20 minutes. The plate was washed 5 times and blotted with absorbent paper. Subsequently, 100 µL of a color-developing agent was added to each well and incubated in the dark for 20 minutes. After incubation, 50 µL of stop solution was added, and the absorbance was measured within 5 minutes on a microplate reader. The concentration of IL-1α in the sac fluid was calculated using the ELISA software.

### Data analysis and statistical methods

Analysis of variance (ANOVA) was used for comparing RS_0–3_, RS_3–6_, and RS_6–12_ data (Tukey's test was used for pair comparison). The correlation amongst RS, intracapsular pressure, preoperative volume, age, IL-1α concentration, and other factors was analysed using linear correlation, and the test standard was α = 0.05.

## Results

### Relevant data measurement results

Amongst the 50 patients, the maximum, minimum, and mean preoperative volume was 46.88 cm^3^, 11.30cm^3^, and 29.08cm^3^, respectively. Regarding RS_0–3_, RS_3–6_, and RS_6–12_, the maximum value was 58.84%, 32.79%, and 10.36%, respectively; the minimum value was 19.83%, 4.32%, and 0.71%, respectively; and the mean value was 45.71%, 17.46%, and 3.933%, respectively. The maximum, minimum, and mean intracapsular pressure was 49 mm Hg, 12 mm Hg, and 24.36 mm Hg, respectively (Table).

**Table 1:** Measurement of intracapsular pressure, age, preoperative cystic cavity volume, and calculation results of rate of shrinkage (RS) in each stage

Serial number	Age, y	Preoperative cystic cavity volume, cm^3^	Intracapsular pressure, mm Hg	RS_0-3_ (%)	RS_3-6_ (%)	RS_6-12_ (%)
1	24	34.5725	24	51.6	17.58	9.45
2	35	14.59941	27	34.04	16.69	5.04
3	18	20.56367	19	54.82	16.06	7.48
4	29	11.42215	49	57.37	27.88	0.71
5	22	19.74696	24	53.86	23.26	1.72
6	20	22.27845	27	58.84	19.73	7.17
7	40	39.14261	13	46.07	19.38	6.02
8	55	33.15642	16	43.25	12.88	4.36
9	66	25.4033	26	44.18	18.76	3.24
10	44	16.87094	42	19.83	4.32	1.44
11	33	23.59262	28	39.7	7.13	4.37
12	41	27.26429	27	56.94	23.95	3.87
13	39	23.75397	49	30.82	12.45	3.94
14	26	27.20397	27	41.55	32.79	7.23
15	26	30.26364	17	48.3	24.43	5.76
16	18	12.73684	29	32.52	9.25	1.23
17	19	12.66061	32	35.64	8.25	2.57
18	22	17.50719	29	40.8	29.88	4.21
19	26	11.30181	30	35.82	15.62	3.84
20	27	45.67596	17	55.51	15.72	2.38
21	18	17.45844	22	43.27	15.31	3.79
22	24	18.56191	30	34.28	9.75	3.64
23	27	37.51607	17	54.09	11.56	2.57
24	37	31.09398	20	52.39	10.45	2.66
25	19	46.88107	12	54.82	20.44	4.47
26	25	29.8542	16	45.25	20.33	3.25
27	20	29.88421	20	50.25	18.52	4.21
28	44	14.521	32	38.82	17.63	1.23
29	25	32.5212	17	55.25	22.34	2.14
30	20	25.25456	25	48.39	19.35	3.52
31	20	28.52354	25	50.23	14.65	4.21
32	22	30.2457	17	52.16	20.52	1.23
33	48	18.25412	29	39.87	18.25	2.32
34	37	24.5631	27	45.26	14.32	1.25
35	24	30.2545	18	52.62	25.31	2.65
36	22	35.6245	16	55.63	24.11	3.65
37	32	20.25415	24	45.27	15.62	4.78
38	35	20.5647	25	48.25	20.25	5.28
39	35	19.5687	26	34.24	12.52	4.35
40	21	36.5415	15	56.32	17.65	6.25
41	30	27.54182	20	43.25	20.32	10.36
42	22	28.51342	18	52.51	20.46	2.36
43	25	21.57841	24	48.65	18.52	3.56
44	32	19.51271	27	39.65	13.25	4.25
45	20	35.2581	20	50.36	19.85	3.25
46	21	40.65412	18	56.23	21.63	6.21
47	30	17.51842	30	35.26	10.11	5.12
48	32	18.51736	28	36.25	10.85	4.32
49	22	26.57132	18	50.32	23.72	2.53
50	35	14.58715	30	35.12	9.25	1.23
Mean value	29.08	25.36	24.36	45.71	17.46	3.933

### RS at different time points after decompression

**Fig. 3:**
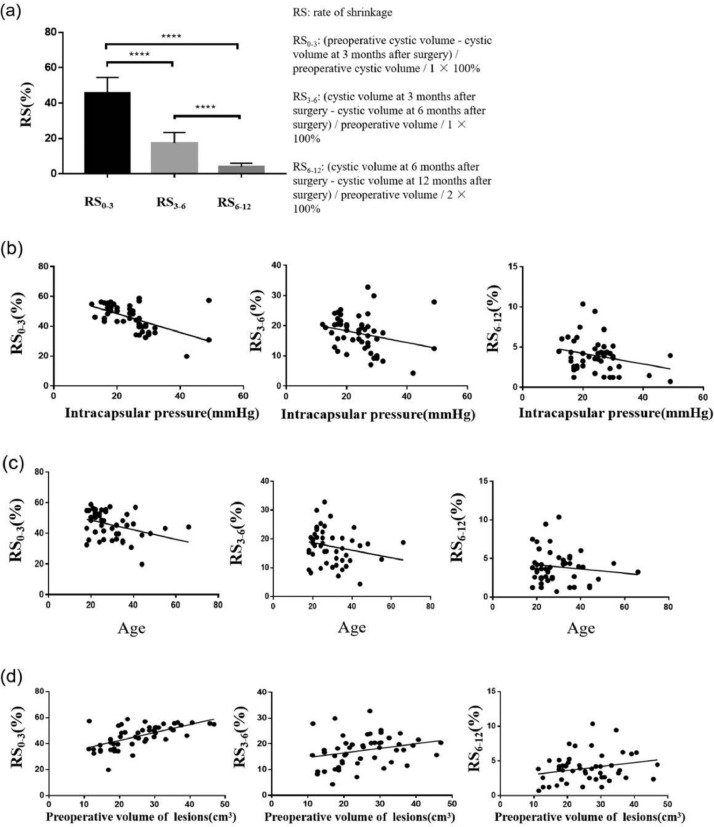
Rate of shrinkage (RS) at each stage and the relationship amongst intracapsular pressure, age, and preoperative cystic volume in adults. A, Comparison amongst RS_0-3_, RS_3-6_, and RS_6-12_. B, Relationship between RS and intracapsular pressure at each stage. C, Relationship between RS at different stages and age. D, Relationship between RS at each stage and the preoperative cystic volume in adults.

ANOVA was used to analyse RS at different time points after decompression (Tukey's test was used for pairwise comparison). Results indicated that RS_0–3_ was the fastest, followed by RS_3–6_, and RS_6–12_. The difference amongst RS_0–3_, RS_3–6_, and RS_6–12_ was statistically significant (Figure 3A).

### Relationship between RS at different time points and intracapsular pressure

The relationship between RS and intracapsular pressure at each stage was analysed using linear correlation. RS_0–3_ was negatively correlated with intracapsular pressure (*r* = −0.6326, n = 50, *P* < .0001), that is, the higher the intracapsular pressure, the slower the rate of volume reduction during the first 3 months after surgery. No correlation between RS_3–6_ and intracapsular pressure (*r* = −0.1936, n = 50, *P* > .05) and between RS_6–12_ and intracapsular pressure (*r* = −0.06638, n = 50, *P* > .05) was observed (Figure 3B).

### Relationship between RS at different time points and age

The correlation between RS at different time points and age was analysed using linear correlation. A negative correlation was observed between RS_0–3_ and age (*r* = −0.3112, n = 50, *P* < .01); that is, the younger the age, the faster the rate of sac volume shrinkage during the first 3 months after surgery. However, no correlation between RS_3–6_ and age (*r* = −0.1303, n = 50, *P* > .05) and between RS_6–12_ and age (*r* = −0.02757, n = 50, *P* > .05) was observed. That is, the effect of age on osteogenesis rate after fenestration only existed during the first 3 months after surgery (Figure 3C).

### Relationship between RS at different time points and preoperative lesion volume

The correlation between RS at different time points and preoperative lesion volume was analysed using linear correlation. A positive correlation was observed between RS_0–3_ and preoperative lesion volume (*r* = 0.611, n = 50, *P* < .0001); that is, the larger the preoperative lesion volume, the faster the lesion volume decreased during the first 3 months after surgery. However, no correlation between RS_3–6_ and preoperative lesion volume (*r* = 0.1781, n = 50, *P* > .05) and between RS_6–12_ and preoperative lesion volume (*r* = 0.05715, n = 50, *P* > .05) was observed. That is, the effect of preoperative lesion volume on osteogenesis rate after fenestration only existed during the first 3 months after surgery (Figure 3D).

### Measurement of IL-1α concentration

**Fig. 4:**
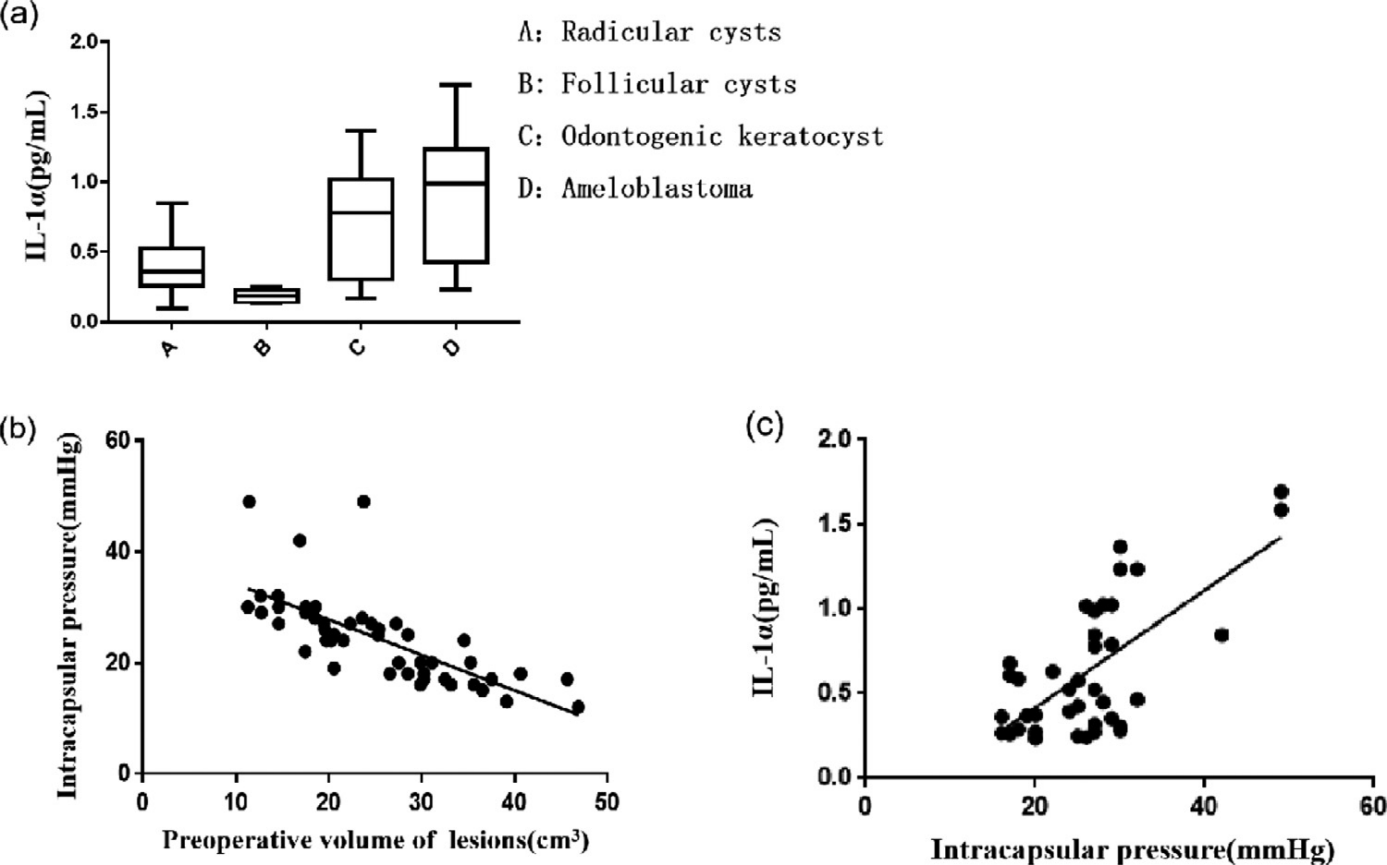
Content of interleukin-1α (IL-1α) and its relationship with intracapsular pressure and preoperative volume of the cystic cavity. A, IL-1α concentration in the cystic fluid of different pathological types. B, Relationship between intracapsular pressure and preoperative volume of the cystic cavity. C, Relationship between IL-1α concentration and intracapsular pressure in the cystic fluid.

The highest and lowest IL-1α concentrations in the radicular cystic fluid of 22 patients were 0.8456 pg/mL and 0.09995 pg/mL, with an average of 0.3825 pg/mL. The highest and lowest IL-1α levels in the radicular cystic fluid of 6 patients were 0.2485 pg/mL and 0.1362 pg/mL, with an average of 0.184 pg/mL. The highest and lowest IL-1α levels in the cystic fluid of 11 patients with odontogenic cystic keratoma were 1.367 pg/mL and 0.1544 pg/mL, with an average of 0.6242 pg/mL. The highest and lowest IL-1α levels in the cystic fluid of 11 patients with ameloblastoma were 1.694 pg/mL and 0.2354 pg/mL, with an average of 0.9227 pg/mL (Figure 4A).

### Relationship between intracapsular pressure and preoperative lesion volume

The correlation between intracapsular pressure and preoperative lesion volume was analysed using linear correlation. A negative correlation between intracapsular pressure and preoperative lesion volume (*r* = −0.6384, n = 50, *P* < .001) was observed; that is, the greater the preoperative lesion volume, the lower the intracapsular pressure (Figure 4B).

### Relationship between IL-1α concentration in cystic fluid and intracapsular pressure

The correlation between IL-1α concentration and intracapsular pressure was analysed using linear correlation. A significant positive correlation was observed between IL-1α concentration in the cystic fluid and the intracapsular pressure (*r* = 0.03477, n = 50, *P* < .0001); that is, the higher the intracapsular pressure, the higher the concentration of IL-1α in the cystic fluid (Figure 4C).

## Discussion

Jaw cystic lesions can be developmental or inflammatory in origin, and lesion progression is closely related to intracapsular pressure and intracapsular-related factors.^13^, ^14^, ^15^ Oka et al reported that positive pressure enhanced the expressions of IL-1α mRNA and protein in odontogenic keratocyte epithelial cells and increased the secretion of matrix metalloproteinase (MMP)-1, MMP-2, MMP-3, and PGE2 in coculture; these factors are closely related to osteoclasts and are crucial for jaw cystic disease progression.^16^ In our study, a significant positive correlation was observed between IL-1α concentration in the sac fluid and the intracapsular pressure. We observed that higher intracapsular IL-1α concentration resulted in poor osteogenesis after fenestration. When IL-1α concentration was low, osteogenesis was better after decompression. Therefore, we hypothesised that the bone wall gets severely damaged under the condition of high IL-1α concentration for a long time. Even if the cystic pressure was released by fenestration and IL-1α concentration was reduced, the recovery of osteogenesis ability was relatively slow after surgery. However, when IL-1α concentration was low, peripheral bone damage was less severe. At this time, although IL-1α concentration decreased only a little after fenestration, the osteogenesis ability was still strong. Of course, IL-1α concentration in the cystic fluid is related not only to the intracapsular pressure but also to the type of jaw cystic lesions. The sample size should be expanded in future studies to overcome the drawback of the uneven distribution of cystic lesions. Previous studies have reported that the pressure in the jaw cyst was negatively correlated with the cyst volume, which was consistent with the results of this study. However, the relationship between cyst pressure and osteogenesis after surgery has not been reported yet. In this study, intracapsular pressure affected the degree and speed of osteogenesis during 3 months after surgery, and the reasons should be further explored. Zhang et al reported that negative pressure could promote bone regeneration by promoting osteogenic differentiation.^17^ Some studies have used decompression combined with negative pressure suction and pressure balls or other devices to deal with negative intracapsular pressure for a certain time every day, with good results.^18^^,^^19^

Some studies have reported that the longer the window decompression time, the greater the decrease in cystic lesion volume, and the higher the initial cystic lesion volume, the faster the recovery rate,^20^^,^^21^ which is consistent with the results of this study. In addition, we studied the osteogenesis rate at different time points after fenestration. The fastest recovery of cystic lesions was observed between 0 and 3 months after surgery, whereas the recovery rate between 6 and 12 months was slow. Therefore, the prognosis of fenestration can be approximately determined according to the changes in cystic lesion volume during 3 months after surgery. If the recovery degree is low during the first 3 months, early surgical treatment should be performed. Early surgical treatment may be considered if critical anatomical structures are already affected because of the slow recovery rate between 6 and 12 months after fenestration. However, we determined that the timing of the second operation depends not only on the amount of new bone but also on the bone density. If the bone density is too low, the new bone may be damaged during the second operation. A small number of patients undergoing early surgery after decompression were included in this study; therefore, the sample size should be expanded to study the changes in bone density and determine the optimal timing of surgery. In some patients, maxillary bone cyst damages maxillary sinus structure or causes facial asymmetry due to occurrences, such as buccal dilation. These changes can be avoided by restoring maxillary sinus structure after window decompression or alleviating buccal bone wall dilation. Inferior alveolar nerve transposition was also observed due to cystic changes in the mandible; however, a new inferior alveolar nerve tube was formed after fenestration.

## Conclusions

Cystic lesion volume reduction was fastest during the first 3 months after decompression surgery. Intracapsular pressure, patient age, and initial cavity volume were the factors that affected the rate of volume reduction during the first 3 months after surgery. The effectiveness of decompression can be predicted by intracapsular pressure, preoperative volume, and age when decompression is selected clinically. However, if important structures have been affected, early secondary curettage should be considered 6 months after surgery. A significant positive correlation was observed between IL-1α concentration in cystic fluid and the intracapsular pressure, and both play an important role in osteogenesis after decompression.

## Author contributions

Xiaomeng Song and Xianbin Xiong contributed to the conception and design of the study and analysis and interpretation of data. Changshun Li, Li Guo, Yuanyuan Wu, and Yi Wang contributed to data collection and manuscript revision.

## Funding

This work was supported by the National Natural Science Foundation of China (81772887), Jiangsu Provincial Medical Innovation Team (CXTDA2017036), the Priority Academic Program Development of Jiangsu Higher Education Institutions (PAPD, 2018-87).

## Conflict of interest

None disclosed.
